# Comparison of Mathematics Problem-Solving Abilities in Autistic and Non-autistic Children: the Influence of Cognitive Profile

**DOI:** 10.1007/s10803-022-05802-w

**Published:** 2022-11-01

**Authors:** Irene Polo-Blanco, Paula Suárez-Pinilla, Juncal Goñi-Cervera, Marta Suárez-Pinilla, Beatriz Payá

**Affiliations:** 1 Departamento de Matemáticas Estadística y Computación, Facultad de Ciencias, Universidad de Cantabria, Santander, Spain; 2 Hospital Universitario Marqués de Valdecilla, Santander, Spain; 3 CIBERSAM. Centro de Investigación Biomédica en Red en Salud Mental, Madrid, Spain; 4 Grupo de Psiquiatría. IDIVAL: Instituto de Investigación Valdecilla, Santander, Spain; 5 Department of Neurodegenerative Diseases, Queen Square Institute of Neurology, UCL, London, UK; 6 Departamento de Medicina y Psiquiatría, Universidad de Cantabria, Santander, Spain

**Keywords:** Autism spectrum disorder (ASD), Mathematic learning, Mathematical problem solving, Strategies executive function, Theory of mind

## Abstract

This study examines relationships between mathematical problem-solving performance (in terms of strategies used and accuracy) and the main cognitive domains associated with mathematical learning (i.e. executive functions, verbal comprehension and social perception) of children with and without autism spectrum disorder (ASD and non-ASD resp.). The study involved 26 ASD and 26 non-ASD children without intellectual disabilities, between 6 and 12 years old, matched by sex, age and school (grade and classroom). The results show a higher percentage of ASD children with problem solving difficulties than non-ASD (57% vs. 23% resp.). Poor performing ASD children showed comparatively lower scores in inhibition, theory of mind and verbal comprehension. Implications for the design of mathematical interventions for ASD students are discussed.

In recent decades, there has been a considerable increase in the number of children with autism spectrum disorders without intellectual disability (from now on, ASD) who attend general education classrooms (McDonald et al., 2019). Because of this, there has also been a growing interest in studying the academic performance of ASD children, and in particular their mathematical performance. Even though the data from some studies show that a considerable percentage of people with ASD without intellectual disability have special mathematical skills (Baron-Cohen et al., 2007; Chen et al., 2019), the findings of low mathematical achievement in this population are much more consistent (Bullen et al., 2020; Estes et al., 2011; Griswold et al., 2002). The main difficulties are observed when faced with mathematical word problems that contain indirect language, superfluous information or require several steps to solve (Bae et al., 2015). Some studies show that, although ASD students without intellectual disability may use the same strategies as students without ASD diagnosis (from now on, non-ASD) when solving mathematical problems, in ASD students more rudimentary strategies like those based on drawing and counting persist (Alderson-Day, 2014; Bae et al., 2015; Goñi-Cervera et al., 2022; Polo-Blanco et al., 2019), while non-ASD children soon exhibit progress to more efficient strategies that require higher level of abstraction, like arithmetic operations (Brissiaud & Sander, 2010; Ivars & Fernández, 2016; Mulligan & Mitchelmore, 1997; Rodríguez Marcos et al., 2008; Siegler, 1988).

Several studies have delved into the relationship between mathematical problem solving performance and cognitive abilities in children with ASD (Bullen et al., 2020; Gonzalez-Gadea et al., 2014). Although the results are heterogeneous, a low executive functioning profile is one of the most consistently replicated findings in this population (McLean et al., 2014; Merchán-Naranjo et al., 2016) and have been linked to poorer performance in mathematical word problem solving (Barnett & Cleary, 2015; Swanson & Beebe-Frankenberger, 2004). Executive functions encompass a wide range of cognitive skills aimed at achieving goals and planning. Within them, several cognitive processes have been reported to be altered in this population, such as response inhibition (Sanderson & Allen, 2013), cognitive flexibility (Yasuda, 2014) and working memory (Bennetto et al., 1996).

Verbal comprehension, which has been most closely related to problem-solving abilities (Alderson-Day, 2014), is also frequently altered in this population, as evidenced by a characteristic pattern in verbal comprehension tests, with high scores in the similarity subtests, but low scores in the comprehension subtests (Mayes & Calhoun, 2008). Research with neurotypical population has shown an impact of theory of mind (hereinafter, ToM) on mathematics competence (Lecce et al., 2014). Moreover, ToM has been claimed to be particularly important when performing scientific and mathematical problems, especially in tasks that are presented verbally (Lockl et al., 2017). Other authors have shown relationships between ToM and problem-solving strategies from as early as preschool age (Sperling et al., 2000). The above studies focus on the neurotypical population, and to our knowledge there is no research that examines the relationship between ToM and problem-solving abilities in ASD children.

Given that the current study focuses on Spanish-speaking children, it should be noted that some of the research works mentioned above (e.g., Ivars & Fernández, 2016; Merchán-Naranjo et al., 2016; Polo-Blanco et al., 2019 and Rodríguez Marcos et al., 2008) have also been carried out with Spanish-speaking students. Regardless of the participants' language, previous studies that focus on ASD children are heterogeneous in terms of the mathematical competencies studied: some focus on creativity and mathematical thinking domains (Hetzroni et al., 2019), others explore computational and arithmetic skills (Dubischar-Krivec et al., 2009), and geometry and representation of objects (Dixon et al., 2016), and a few focus on approaching and solving mathematical problems (Bae et al., 2015; Oswald et al., 2016). However, to date, no studies have been published that explore the relationship between the accuracy when solving mathematical problems, the level of abstraction of the strategies used in the solving process, and cognitive domains in ASD children without intellectual disability.

Based on this, our first hypothesis is that young school-age ASD children (6–12 years old) without intellectual disability will use more rudimentary strategies (i.e., with a lower level of abstraction, like those based on drawing) to solve mathematical problems compared to the non-ASD population. Second, we hypothesize that ASD children will exhibit greater difficulties solving mathematical problems compared to non-ASD children, resulting on a lower proportion of accurate responses, presumably in relation with the first hypothesis. Lastly, we expect poorer mathematical performance to be associated with worse functioning in such cognitive domains involved in the mathematical problem-solving process (executive functions, verbal comprehension and social perception) in both groups of children. To test these hypotheses, the purpose of this work is to study mathematical performance, measured through the level of abstraction of strategies used during the mathematical problem-solving process and the accuracy of responses, both in ASD and non-ASD children without intellectual disability. We also seek to determine if there is any association between the level of abstraction of the strategies used, the accuracy of responses in solving problems and the main cognitive domains associated with mathematical performance, such as executive functions (response inhibition, cognitive flexibility and working memory), verbal comprehension and social perception (affect recognition and ToM).

## Methods

### Participants

Participants in the study included 26 children in the ASD group (23 males and 3 females, mean age 9.35) and 26 children in the non-ASD group (23 males and 3 females, mean age 9.41). The ASD sample was recruited from different health, social and educational resources that care for individuals with autism in the Spanish region of Cantabria. These resources include child psychiatry and pediatric outpatient clinics, family associations, and school counseling personnel. The participants were recruited between July 2019 and February 2021. The inclusion criteria were: (1) being diagnosed with ASD and absence of another psychiatric comorbidity (including absence of other neurodevelopmental disorder: attention-deficit/hyperactivity disorder (ADHD), dyslexia… etc.), as per the Diagnostic and Statistical Manual of Mental Disorders (DSM-5) (APA, 2013); (2) FSIQ ≥ 70 as measured by Wechsler Intelligence Scale for Children WISC-V (Wechsler, 2015); (3) being between 6 and 12 years old; (4) obtaining a minimum direct score of 26 points in the Test of Early Mathematics Ability (TEMA-3, Ginsburg & Baroody, 2007). A direct score of 26 corresponds to an equivalent mathematical age of 5 years and 6 months that guarantees the development of a minimum knowledge of additive operations that allow dealing with informal multiplication and division strategies (see later section on problem solving strategies).

Once the criteria for the children of the ASD group were verified, a candidate without autism (from now, non-ASD) of the same sex, age, school and grade was contacted through school counselors or managers. Each non-ASD child was selected from the same classroom of the ASD child already enrolled in the study. The inclusion criteria for the non-ASD group were the same as for the ASD group except for the ASD diagnosis.

After receiving a detailed explanation of the characteristics and purpose of the study, all the parents or legal representatives signed the informed consent document. This study was previously approved by the Cantabria Research Ethics Committee (CEIC).

### Measurement Variables and Instruments

Next, the measures of the study are described. All interviews and test applications were conducted in Spanish.

#### Clinical and Sociodemographic Variables

The sociodemographic variables were collected through a structured interview with the participants and their parents or legal guardians. Socioeconomic status was determined using the Hollingshead-Redlich scale (Hollingshead & Redlich, 1958, 2007) that provides five categories: low (I: rating 8–19), low-middle (II: rating 20–29), middle (III: rating 30–39), high-middle (IV: rating 40–54), and high (V: rating 55–66). The scores are obtained from the level of education and occupation of the parents or guardians (the higher the score, the higher the category level).

Confirmation of ASD diagnosis and ruling out possible associated comorbidities for the whole sample (ASD and non-ASD) was based on DSM-5 criteria, and was performed by a child psychiatrist with extensive experience evaluating ASD children. For ASD children, the diagnosis was confirmed through a clinical evaluation of the child, a review of his/her clinical reports, and a detailed anamnesis with the child’s parents to confirm current or past ASD symptoms and to rule out possible current and past symptoms of associated comorbidities. The same child psychiatrist performed a clinical evaluation of each non-ASD child and a detailed anamnesis with his/her parents to obtain information about his/her developmental history, and to rule out current or past psychiatric symptoms.

#### FSIQ Assessment

Since the Estimated Intelligence Quotient (EIQ) is not a reliable measure in ASD patients (Merchán-Naranjo et al., 2016), the Full Scale Intelligence Quotient (hereinafter, FSIQ) test was administered to both ASD and non-ASD children using the Spanish translation of the WISC-V (Wechsler, 2015). The WISC-V is an instrument that can be applied to children and adolescents between 6 years and 0 months and 16 years and 11 months of age. The WISC-V scale provides scores on the primary indexes of intelligence that reflect intellectual functioning in five different cognitive areas: Verbal Comprehension Index (VCI), Visual Spatial Index (VSI), Fluid Reasoning Index (FRI), Working Memory Index (WMI), and the Processing Speed Index (PSI). This assessment also provides a Full Scale IQ (FSIQ) composite score that represents general intellectual ability. The FSIQ is obtained from the scores of the following seven subtests: Block Design, Similarities, Matrix Reasoning, Digit Span, Coding, Vocabulary and Figure Weights. The FSIQ was evaluated by a clinical psychologist with clinical experience in the ASD population.

#### Mathematical Competence

Mathematical competence was assessed using the TEMA-3 test (Ginsburg & Baroody, 2007), which is designed to evaluate mathematical skills in children. It is a performance test with 72 items that assess formal and informal skills (counting, comparing numbers, mastery of number facts and calculation skills). The test scores range from 0 to 72 and are converted into a mathematical age. Its internal consistency has been reported at 0.90 (Ginsburg & Baroody, 2007) for neurotypical population. The instrument has also been used in previous research with children with intellectual and developmental disabilities (e.g. Vostanis et al., 2021).

#### Mathematical Problem Solving (MPI)

In order to evaluate the ability to solve mathematical problems, the MPI (Mathematical Problem Instrument) questionnaire was administered. The MPI was adapted from the study by Mulligan & Mitchelmore (1997) that focuses on studying the strategies of young school-age children when solving multiplication and division problems. The MPI includes eight arithmetic word problems that cover the different types of multiplication and division problems (Nesher, 1992). This instrument has been previously used to evaluate problem solving performance in children with learning disabilities (Parmar, 2003) and ASD children (Polo-Blanco et al., 2022). The children were given a booklet with the eight problems presented in writing, a pen, and manipulative linking cubes that they could use if they so desired.

In order to analyze the problem-solving strategies, the entire MPI application process was videotaped. A member of the research team, with previous experience in the application and coding of this instrument, transcribed and analyzed the videos. The solutions were classified into four types of strategies, arranged from lowest to highest level of abstraction (Ivars & Fernández, 2016; Mulligan & Mitchelmore, 1997): (1) incorrect strategies: when the participant solves the problem incorrectly, for example through addition and subtraction operations instead of multiplication and division (for example, in the problem: “There are two tables, and four people at each table, how many people are there in total?” a child could incorrectly perform the sum 2 + 4 to solve the problem); (2) direct modeling with counting: when the child solves the problem using drawings or manipulatives (for example, to solve the above problem, a child could draw two tables with four people at each table, and count the number of people providing the correct response: 8); (3) counting strategies: when the child solves the problem by resorting to counting actions without using modeling (for example, to solve the previous problem, the child would add the number of people at each table without the need to draw: 2 + 2 + 2 + 2); and (4) number facts: when the child uses multiplication or division operations to solve the problem (in the previous problem, the child would perform the multiplication that solves the problem: 2 × 4).

In addition, responses were coded, in terms of their accuracy, as correct or incorrect. Incorrect responses occurred either when a strategy of type (1) was used, or when another strategy was used that was executed incorrectly. For example, in the previous problem, if a student performed the multiplication as follows: 2 × 4 = 9, it was coded as strategy type (4) and incorrect result.

All the sessions involving problem solving were videotaped. Interobserver reliability data were collected from all participants. One of the authors coded all of the children’ strategies and performance from both groups (ASD and non-ASD). An experienced mathematics education teacher external to the research team, who was blind to the hypotheses of the study, recoded 30% of the data. These data included an equal split of participants from both groups, with cases selected from all participants, and across all problems. Interobserver agreement was calculated by dividing the number of agreements by the number of agreements plus disagreements and multiplying by 100. The mean interobserver reliability agreement for strategy categorization was 97% (Cohen’s *kappa* 0.96) and 99% (Cohen’s *kappa* 0.98) for children in the ASD and non-ASD groups, respectively. The mean interobserver reliability agreement for solution accuracy was 100% (Cohen’s *kappa* 1) for children in both groups.

For the data analysis, we considered two aspects of the MPI: first, the type of strategy according to the level of abstraction, and second, the accuracy of the attained results. The level of abstraction of the strategy was evaluated through examining the median of the eight strategies used by each child to solve the MPI. The median was deemed the most stable statistic to reflect the type of strategy used by each child, since it represents the intermediate value of the set of observed strategies and is less affected by outliers. For example, in the resolution of the eight problems of the questionnaire, a participant could employ the following strategies (1,4,4,4,4,4,4,4). In this case, the median would be 4 and would be representative of performance, since having used an incorrect strategy (1) only once, would probably be due to an oversight.

The accuracy, defined as participant´s percentage of correct responses, was treated both, as a continuous (ranged between 0 and 1), and categorical variable (in this case, hereinafter called accuracy level) and binned into 4 levels: ≤ 25%, 26–50%, 51–75% and > 75% of correct responses. Moreover, those children who obtained ≤ 25% correct answers were classified as poorer performers, with respect the rest of children (with > 25% correct responses). General analyses on accuracy employed the variable in continuous form, while the purpose of the binning was both descriptive (to better show the distribution of performance levels in ASD and non-ASD groups), and also to provide a further focus on the subset of poorer performers.

#### Neurocognitive Variables

For the neurocognitive assessment, subtests from the Neuropsychological Assessment Battery (NEPSY-II) were administered. The NEPSY-II battery has been developed for the neuropsychological evaluation of children from 3 to 16 years old, and has been validated in both general and special populations, including ASD (Korkman et al., 2007).

The NEPSY-II battery covers six cognitive domains that include different subtests. It allows the evaluator to apply the entire instrument or to select from the different subtests those that best fit the assessment objective. Only four subtests were used for this study: two subtests corresponding to the domain of the executive functions, namely: (1) response set (i.e., cognitive flexibility), which assesses the ability to change and maintain a new pattern of responses, and (2) inhibition, which assesses the ability to inhibit automatic answers in favor of another type of answer, and the ability to switch between different response types; and two subtests corresponding to the domain of social perception, namely: (1) affect recognition, which assesses the ability to distinguish common facial emotions; and (2) ToM, which assesses the ability to comprehend other people’s perspectives, intentions, and beliefs. Scaled scores (mean = 10, standard deviation = 3) were used for the analysis, with higher scores indicating better performance.

The internal consistency of the NEPSY-II has been widely studied in both general and special samples, including ASD. For the general sample, the reliability coefficients for the four subsets used in this study were: 0.94 (response set), 0.92 (inhibition), 0.78 (affect recognition) and 0.70 (ToM) (Korkman et al., 2007). Reliability coefficients for the special sample for the subsets inhibition, affect recognition and ToM were respectively: 0.90, 0.84 and 0.79 (Korkman et al., 2007).

The working memory and verbal comprehension subtests of the WISC-V were also applied, respectively, to assess working memory as an executive function and the verbal comprehension domain. In particular, the Working Memory Index (WMI) and the Verbal Comprehension Index (VCI) were considered.

The internal consistency of the Spanish adaptation of the WISC-V has been studied with the reliability quotient of the FSIQ being 0.95 and the indices offer reliability coefficients that vary between 0.88 and 0.93 (for the Spanish typing sample). There are also North American studies of the validity (Stephenson et al., 2021) and reliability of the WISC-V in the ASD population (Wechsler, 2015) providing reliability quotients for the main tests varying between 0.86 and 0.97.

### Statistical Analysis

#### Description of the Sample

The clinical and sociodemographic description of the sample was performed by calculating frequencies and percentages for the categorical variables, and means and standard deviations (*SD*) for the continuous variables. Such descriptive statistics were computed separately within the ASD and non-ASD groups, and the potential between-group differences were ascertained by Chi-square test (χ2) for categorical variables, and Student's t-test (*t*) or Mann–Whitney's U for continuous variables. The latter (U) was preferred when normal distribution was ruled out for the continuous variable in question, according to the Kolmogorov–Smirnov test. Cronbach's α was used to assess the reliability of the employed scores, namely FSIQ, NEPSY, TEMA-3 and MPI. Reliability was calculated separately for ASD and non-ASD groups.

#### Mathematical Performance (Strategy and Accuracy) as a Function of ASD Status

Our first and second hypotheses supposed that ASD children would exhibit poorer mathematical performance, showed in more rudimentary (i.e., less abstract) problem-solving strategies (first hypothesis) and in lower proportion of accurate responses (second hypothesis). These two hypotheses were investigated in parallel through the same statistical tools, only varying in the variable of interest, namely MPI (strategy) and proportion of correct responses (accuracy).

First, we aimed to ascertain overall differences between ASD and non-ASD groups by running two independent *t*-tests: on strategy (median MPI) and accuracy (continuous variable scored out of 1). As explained above, accuracy was also binned into levels (for descriptive purposes and further focus on the poorer performers); potential differences between ASD and non-ASD groups were in this case ascertained by Fisher´s exact test, which was preferred to χ2 test due to the small sample size and expected frequencies per group.

Multivariate analyses were carried out by general linear models (GLM) of covariance analysis (ANCOVA), wherein the dependent variables were median MPI and accuracy: thus, two different models were built for the two hypotheses in question. The following predictors were included in each model: group (ASD/non-ASD) as a fixed factor, and chronological age and those variables wherein significant differences were previously obtained between ASD and non-ASD groups (mathematical age and FSIQ) as continuous covariates. Furthermore, the interaction of each continuous covariate with group was included, in order to examine whether ASD status modulated the effect of age and FSIQ on mathematical strategy and accuracy.

Apart from these analyses on the entire sample, we sought to examine between-group differences within comparable FSIQ levels: with this aim, the sample was stratified into three levels, following the current Wechsler FSIQ classification: < 90 (low average or less); 90–109 (average), and > 110 (high average or superior), and the above-described multivariate analyses were repeated within the level of average FSIQ (90–109). Multivariate analysis could not be carried out within the other two levels given their small sample size.

#### Relationship of Mathematical Strategy and Accuracy in ASD and Non-ASD Groups

A subsidiary aspect of our second hypothesis postulated that the lower accuracy in the ASD group might be in relation with the poorer strategy deployed in problem solving. We surmised that such potential association could be observed especially in the poorer performers (with ≤ 25% correct responses) compared with the rest of children, and that it may behave differently in ASD and non-ASD groups (e.g., due to potential compensation through other cognitive domains). Thus, an ANCOVA was repeated for each group (ASD/non-ASD) in which MPI median was used as dependent variable. The accuracy level (binned into ≤ or > 25%) was regarded as a fixed factor, while the chronological age, mathematical age and FSIQ were considered as covariates.

#### Mathematical Strategy and Various Neurocognitive Domains in ASD and non-ASD Groups

Finally, our third hypothesis proposed an association between scores in different neuropsychological domains (executive functions, verbal comprehension and social perception) and mathematical performance. In addition, we considered the possibility that such association may be modulated by ASD status. We approached this question through three different analyses.

First, a possible bivariate correlation between the abstraction of mathematical strategy (median MPI) and each neuropsychological variable was investigated separately within ASD and non-ASD groups, using Spearman´s correlation coefficient (ρ).

Second, several ANCOVA-GLMs were built, with MPI median and accuracy as dependent variables, and group (ASD/non-ASD), chronological age, mathematical age and FSIQ, as well as the considered neuropsychological domains (included one by one in separate models) as predictors, and also the interaction between ASD status and neuropsychological domains.

Third, in order to investigate potential associations focused in the group of poorer performers, an independent ANCOVA was built for each group (ASD/non-ASD), in which scores of neuropsychological tests were considered as dependent variables, whereas accuracy level (binned into ≤ or > 25%) was regarded as a fixed factor, while chronological age, mathematical age and FSIQ were covariates. All the analyses were conducted with the SPSS 28.0 statistical package (IBM, 2021), and significance was established with a *p* value ≤ 0.05. All comparisons of GLM-ANCOVAS were Bonferroni corrected.

## Results

### Description of the Sample

Initially, a sample of 38 children was recruited for the ASD group. All children had previously been evaluated and diagnosed in mental health units. An experienced child psychiatrist reviewed each child’s records, and confirmed the diagnostic criteria for ASD and the absence of comorbidities through parental interview and patient evaluation, based on DSM-5 criteria. Of the initial 38 children, 12 were excluded because they did not satisfy the inclusion criteria. Specifically, two children were excluded for not meeting the DSM-5 diagnostic criteria for ASD, three for presenting a comorbidity with ADHD, six for presenting an FSIQ < 70, and one for not reaching the required cut-off point of mathematical competence as per TEMA-3. In the end, 26 children comprised the ASD group, and the same number was recruited for the non-ASD group (23 males and 3 females in each group). As shown in Table 1, there were no significant between-group differences in terms of chronological age, sex, and parental socioeconomic status. However, the ASD group had significantly lower mathematical age, obtained from TEMA-3 score (*t* (50) = − 2.89, *p* = 0.006, *d* = − 0.81) and lower FSIQ (*t* (50) = − 2.34, *p* ≤ 0.001, *d* = − 1.06) than the non-ASD group (Table 1). Because of this, mathematical age and FSIQ were considered as covariates in the subsequent analyses. Regarding psychopharmacological medication, only one ASD child was taking an antipsychotic (risperidone). No patient in the non-ASD group was taking any psychotropic drugs.

**Table 1:** Sociodemographic and clinical data and mathematical competence

	ASD (*N* = 26)	non-ASD (*N* = 26)	Statistics	*p*	Effect size
Sex (males)	23 (88.4%)	23 (88.4%)	χ^2^(2) = 0.00	1.000	Φ = 0.00
Age (years)	9.35 *(2.06)* [6.25–12.92]	9.41 *(1.96)* [6.25–12.42]	t (50) = − 0.10	.922	*d* = − 0.03
Parental SES			χ^2^(4) = 4.56	.336	V = 0.30
V (High level)	5 (19.2%)	9 (34.6%)			
IV (High-middle level)	8 (30.8%)	8 (30.8%)			
III (Middle level)	8 (30.8%)	3 (11.5%)			
II (Low-middle level)	4 (15.4%)	3 (11.5%)			
I (Low level)	1 (3.8%)	3 (11.5%)			
Mathematical competence					
TEMA-3 score	54.00 *(13.15)* [26- 72]	62.81 *(10.19)* [41–72]	*t* (50) = -2.70	.009**	*d* = − 0.75
Mathematical age	7.56 *(1.10)* [5.50–9.00]	8.38 (*0.93*) [6.50–9.00]	*t* (50) = − 2.89	.006**	*d* = − 0.81
WISC-FSIQ	89.88 (*11.78*) [70–115]	102.00 (*10.98*) [81—130]	*t* (50) = − 3.84	< .001**	*d* = − 1.06
Executive functions					
Working memory	89.85 *(12.47)* [69–117]	100.54 *(11.56)* [79–122]	*t* (50) = − 3.19	.002**	*d* = − 0.89
Response set	7.62 *(3.44)* [2—14]	8.71 *(3.04)* [4–14]	*t* (50) = − 1.09	.281	*d* = − 0.34
Inhibition	6.62 *(3.31)* [1–14]	9.31 *(3.80)* [1–15]	*t* (50) = − 2.72	.009**	*d* = − 0.75
Verbal comprehension	89.81 (*19.29)* [45–130]	104.96 (*11.84)* [86–133]	*t* (50) = − 3.41	.001**	*d* = − 0.95
Social perception					
Affect recognition	7.50 *(3.34)* [1–18]	9.92 *(1.94)* [7–13]	*t* (50) = -3.20	.002**	*d* = − 0.89
Theory of mind	16.31 *(6.79)* [0–26]	22.54 *(2.52)* [17–26]	*U* = 126.50	< .001**	η^2^ = 0.29

For categorical variables, the absolute frequency (and %) is provided; continuous variables present the mean *(SD)*. The p value pertains to statistical comparisons between both groups (ASD vs non-ASD): χ^2^ for categorical variables and *t*-test for quantitative variables

The employed effect sizes are: Cohen´s D for normally distributed continuous variables, η^2^: for non-normally distributed continuous variables, Phi effect size for dichotomic categorical variables, and Cramer´s V for categorical variables with more than 2 categories

*ASD* Autism Spectrum Disorders without intellectual disability, *d* Cohen´s D; η^2^ Eta squared effect size, *Φ* Phi effect size, *SD* Standard deviation, *SES* socioeconomic status assessed with the Hollinshead Scale, *TEMA* Test of early mathematics ability, *V* Cramer´s V, *FSIQ* Wechsler intelligence scale for children-intelligence quotient

*p ≤ .05; **p ≤ .01

Regarding neurocognitive variables (see Table 1), significant differences were found in some executive functions. Specifically, the ASD group had lower scores in working memory and inhibition. However, no significant differences were found in terms of response set (cognitive flexibility). Significantly lower scores were also observed in the ASD group in verbal comprehension. Regarding the domain of social perception, ASD children scored significantly lower both in affect recognition and in ToM. After adjustment by FSIQ, age and mathematical age, only verbal comprehension and ToM remained significantly different between ASD and non-ASD groups.

The reliability of the employed test scores for ASD and non-ASD groups, respectively, was as follows: FISQ: α = 0.62/0.69; TEMA-3: α = 0.89/0.71; MPI: α = 0.93/0.84; and NEPSY: α = 0.89/0.70.

### Mathematical Performance (Strategy and Accuracy) as a Function of ASD Status

As shown in Table 2, ASD and non-ASD groups showed no significant differences in the two considered aspects of mathematical performance, namely strategy (level of abstraction as indicated by the median MPI score: *t* (50) = 0.44, *p* = 0.339, *d* = − 0.27) and accuracy (*t* (50) = − 1.62, *p* = 0.112, *d* = − 0.45). However, considering binned accuracy level (percentage of correct responses: ≤ 25%, 26–50%, 51–75%, > 75%), the distribution of ASD and non-ASD children within each of the four bins was significantly different according to Fisher's exact test: *Fisher´s* = 8.57, *p* = 0.034, *V* = 0.41. As shown in Table 2, this imbalance was due to an excess of poorest performers (≤ 25% correct responses) of the ASD group: 57%, for only 23% of the non-ASD group.

**Table 2:** Mathematical problem solving in ASD and non-ASD students

MPI median score	ASD (*N* = 26)		Non-ASD (*N* = 26)		Statistics	*p*	Effect size
	1.98 (*1.34*), range:1–4		2.35 (*1.38*), range: 1–4		*t* (50) = 0.44	.339	*d* = − 0.27
Accuracy (out of 1)	0.35 (*0.38*), range:0–1		0.50 (*0.28*), range: 0.13–1		*t* (50) = − 1.62	.112	*d* = − 0.45
Level of accuracy	Observed	Expected	Observed	Expected			
0–25%	15 (57%)	10.5 (40.4%)	6 (23%)	10.5 (40.4%)	Fisher’s = 8.57	.034*	*V* = 0.41
26–50%	3 (11.5%)	7 (26.9%)	11 (42.3%)	7 (26.9%)			
51–75%	3 (11.5%)	3.5(13.5%)	4 (15.4%)	3.5 (13.5%)			
76–100%	5 (19.2%)	5 (19.2%)	5 (19.2%)	5 (19.2%)			

For categorical variables, the absolute frequency (and %) is provided; continuous variables present the mean *(SD)*. The p value pertains to statistical comparisons between both groups (ASD vs non-ASD): Fisher´s exact test for categorical variables and t-student for quantitative variables

The employed effect sizes are: Cohen´s D for normally distributed continuous variables and Cramer´s V for categorical variables with more than 2 categories

*ASD* Autism Spectrum Disorder without intellectual disability, *d* Cohen´s D, *MPI* Mathematical Problem Instrument, *SD* Standard deviation, *V* Cramer´s V

*p ≤ .05

For multivariate analysis, chronological and mathematical age, as well as FSIQ, were included as covariates. In the resulting ANCOVA models, ASD status was not a significant predictor of strategy: *F* (1,50) = 0.88, *p* = 0.352, η^2^ = 0.02. Likewise, interactions of group (ASD/non-ASD) with the rest of covariates were not significant, indicating that ASD status did not moderate the effect of age and FSIQ on strategy.

Multivariate analysis on accuracy also yielded no significant difference between groups:* F* (1,50) = 0.37, *p* = 0.546, η^2^ = 0.01. Interactions between ASD status and covariates were not statistically significant.

Subsequently, the dataset was stratified into three levels according to their FSIQ. In the FSIQ level < 90 (i.e., low average or less) there were 12 ASD and 3 non-ASD children. The average-FSIQ level (90–109) was formed by 11 ASD children and 17 non-ASD children. Finally, the FSIQ level ≥ 110 (high average or more) had 2 ASD and 6 non-ASD children. Given the small sample size available for each FSIQ level, multivariate analysis could only be carried out on the average-FSIQ subset. Within this level, ASD children exhibited significantly better strategy [*F* (1,26) = 8.25, *p* = 0.009, η2 = 0.29] and accuracy [F (1,26) = 6.34, *p* = 0.020, η2 = 0.24]. Specifically, adjusted marginal means for strategy/MPI score were 2.18 (*SE* = 0.33) in ASD *vs*. 2.06 (*SE* = 0.24) in non-ASD group. Likewise, proportion of accurate responses had adjusted marginal means of 0.49 (*SE* = 0.06) vs. 0.44 (*SE* = 0.04) for ASD and non-ASD children, respectively. Moreover, significant interactions between group and FSIQ were found for both strategy [F (1,26) = 8.34; *p* = 0.009, η^2^ = 0.29] and accuracy [F (1,26) = 8.65; *p* = 0.008, η^2^ = 0.30]. In both models (strategy and accuracy), the regression coefficient for the main effect of ASD group had positive sign, whereas its interaction with FSIQ was negative (values not shown). This suggests that, within the average-FSIQ level, strategy and accuracy are better in the ASD group, *and* less sensitive to variations in FSIQ than for non-ASD children.

### Relationship of Mathematical Strategy and Accuracy in ASD and non-ASD Groups

ASD children with lower accuracy (≤ 25% of correct responses) also exhibited worse strategy than the rest of the ASD group [*F* (1,24) = 14.59, *p* = 0.001, η^2^ = 0.29]. Adjusted marginal means of MPI were 1.30 (*SE* = 0.22) among poorer performers, *vs.* 2.90 (*SE* = 0.27) for the rest. This indicates that the worst performing ASD children use mostly incorrect strategies (MPI = 1) (e.g. providing a random number as a solution, or performing addition of the data instead of multiplication).

However, in the non-ASD group, we found no significant differences in strategy between the poorer performers and all others [*F* (1,24) = 0.95, *p* = 0.340, η^2^ = 0.04].

### Mathematical Strategy and Various Neurocognitive Domains in ASD and Non-ASD Groups

In the ASD group, significant positive correlations were observed between strategy and the following cognitive domains: response set (Spearman´s ρ = 0.52; *p* = 0.016), inhibition (ρ = 0.44; *p* = 0.024) and ToM (ρ = 0.70; *p* ≤ 0.001). However, in the non-ASD group, strategy was not significantly correlated to any of the cognitive variables studied (Table 3).

**Table 3:** Bivariate correlation between neuropsychological variables and MPI in ASD and non-ASD groups

Neurocognitive variables	ASD (*N* = 26)		Non-ASD (*N* = 26)	
	Spearman´s ρ	*p*	Spearman´s ρ	*p*
Executive function				
Response set	.52	.016*	.26	.252
Inhibition	.44	.024*	.14	.505
Working memory	.30	.141	.17	.407
Verbal comprehension	.34	.086	− .10	.637
Social perception				
Affect recognition	.11	.603	− .07	.746
Theory of mind	.70	< .001**	.28	.164

*ASD* Autism spectrum disorder without intellectual disability, *ρ* Spearman´s coefficient

*p ≤ .05, **p ≤ .01

Multivariate models on strategy and accuracy were run, adding as covariates (beside those described in the above sections) the explored neuropsychological domains relevant to mathematical performance: executive functions, verbal comprehension and social perception. None of these domains showed association with strategy or accuracy. Likewise, there was no significant interaction between such neuropsychological domains and group (ASD/non-ASD). The latter suggested that ASD status did not modulate the relationship between explored neuropsychological domains and mathematical performance.

The lack of interaction seems at odds with the results of the bivariate correlations as reported above (for response set, inhibition and ToM), where the association with strategy (MPI) was present in the ASD group only. We reasoned that specific associations between cognitive scores and mathematical performance could be more easily evidenced if comparison was made between the poorest performers and all the rest, rather than all along the entire range of the continuous variable.

Thus, further analyses were focused on the relation of ASD status and the specific neuropsychological domains, considering the group of children with low performance in mathematical problem-solving. Among the ASD children, the poorer performers (≤ 25% of correct answers) attained lower scores than the rest of ASD children in verbal comprehension [*F* (1,24) = 4.94, *p* = 0.037, η^2^ = 0.19] inhibition [*F* (1,24) = 4.34, *p* = 0.050, η^2^ = 0.17] and ToM [F (1,24) = 5.08, *p* = 0.035, η^2^ = 0.20]. Conversely, for non-ASD children, there were no significant differences between poorer performers and all others regarding any of the explored neurocognitive domains (Table 4).

**Table 4:** Neurocognitive scores based on the accuracy rate groups in ASD and non-ASD students

Neurocognitive variables	ASD (*N* = 26)				Non-ASD (*N* = 26)			
	≤ 25% (*n* = 15) Mean (*SE*)	> 25% (*n* = 11) Mean (*SE*)	*F* (1, 24); *p*	Effect size	≤ 25% (*n* = 6) Mean (*SE*)	> 25% (*n* = 20) Mean (SE)	*F* (1, 24); *p*	Effect size
Executive functions								
Working memory	86.85 (*3.48*)	93.93 (*4.36*)	*F* = 1.12; *p* = .302	η^2^ = 0.05	99.53 (*5.40*)	100.84 (*2.63*)	*F* = 0.04; *p* = .841	η^2^ = 0.00
Response set	6.28 (*1.27*)	8.84 (*1.20*)	*F* = 1.522; *p* = .235	η^2^ = 0.09	10.05 (*1.72*)	8.63 (*0.63*)	*F* = 0.68; *p* = .423	η^2^ = 0.04
Inhibition	4.82 (*1.06*)	9.07 (*1.33*)	*F* = 4.34; *p* = .050*	η^2^ = 0.17	10.78 (*1.67*)	8.86 (*0.81*)	*F* = 0.93; *p* = .346	η^2^ = 0.04
Verbal comprehension	83.81 (*3.32*)	97.98 (*4.16*)	F = 4.94; *p* = .037*	η^2^ = 0.19	105.47 (*4.57*)	104.81 (*2.23*)	*F* = 0.01; *p* = .905	η^2^ = 0.00
Social perception								
Affect recognition	7.78 (*1.15*)	7.12 (*1.44*)	*F* = 0.08; *p* = .706	η^2^ = 0.00	10.09 (*0.95*)	9.87 (*0.46*)	*F* = 0.04; *p* = .844	η^2^ = 0.00
Theory of mind	13.50 (*1.54*)	20.13 (*1.92*)	*F* = 5.08; *p* = .035*	η^2^ = 0.20	23.06 (*0.97*)	22.38 (*0.47*)	*F* = 0.34; *p* = .561	η^2^ = 0.02

*ASD* Autism spectrum disorder without intellectual disability, *SE* Standard error, η^2^ Partial eta squared effect size

*p ≤ .05

## Discussion

In this work, we have examined relationships between mathematical problem-solving performance (in terms of the strategies used and accuracy of responses) and the main cognitive domains associated with mathematical performance in ASD and non-ASD children. Our results point to a higher prevalence of ASD children with problem solving difficulties than non-ASD. Those ASD students who exhibited poorer mathematical performance (i.e., those who obtained ≤ 25 of correct responses) showed lower scores in executive functions, predominantly in working memory and inhibition, as well as in their verbal comprehension and social perception (affect recognition and ToM), compared with the rest of ASD children (i.e., those who attained > 25% of correct responses). These differences were not observed in the group of non-ASD children with poor performance, compared with the rest of non-ASD children. These results are in line with previous works which support a low executive functioning profile in ASD children without intellectual disabilities (Merchán-Naranjo et al., 2016; Ozonoff et al., 1991; Rumsey & Hamburger, 1988) and lower verbal and ToM scores (Loukusa et al., 2018).

Another of our findings of interest is the children distribution based on the accuracy of responses in the MPI test. We found a significantly higher proportion of ASD children (57%) compared to non-ASD children (23%) in the group of poorer performers (group with ≤ 25% correct answers), while non-ASD were more frequent than ASD children in the group with 26–50% correct answers; however, in the groups with the highest performance (success rate of 51–75% and > 75% correct responses), there were no considerable differences in the proportion of ASD compared to the non-ASD children. On the one hand, we found a higher proportion of ASD children among those with greater difficulties solving mathematical problems and, on the other hand, a subgroup of ASD children who seem to exhibit no difficulties in solving mathematical problems, presenting a performance equal to that of the non-ASD group. Contrary to our initial hypothesis, we found no overall differences between the level of abstraction of strategy used to solve mathematical problems in both groups (ASD and non-ASD). This indicates that the strategies used by ASD children when attempting to solve mathematical problems are globally similar to those used by non-ASD ones, which further supports that ASD is not necessarily linked to atypical or abnormal development in mathematics learning. However, when the type of strategy used by ASD and non-ASD children was analyzed based on the accuracy rate of responses obtained in the MPI, we found that the level of abstraction of strategy used differs in poorer performing ASD children compared with the rest of ASD, but curiously not in the non-ASD children. Thus, we observed that the poorer performing ASD children used less elaborate strategies (like those based on drawing or counting) than the rest of ASD children, who used more advanced strategies (like those based on arithmetic operations).

Some previous studies show that low performance in mathematical problem-solving is frequent in ASD population (Bullen et al., 2020; Estes et al., 2011; Griswold et al., 2002). In our case, we found differences in these abilities within the ASD group with no intellectual disability, in line with other authors' findings (Chen et al., 2019; Oswald et al., 2016; Whitby, 2013). However, one should be cautious in drawing conclusions in this regard. First, the difference in context and measures used in other studies does not allow to stablish rigorous comparisons. In addition, the MPI instrument used in this study has not been validated in the ASD population, although as mentioned above, there are precedents in the literature that use this test with similar populations (Parmar, 2003; Polo-Blanco et al., 2022).

Another interesting finding is the positive correlation found between the level of abstraction of the strategy used and three cognitive variables—inhibition, cognitive flexibility and ToM—in the whole group of ASD children, which was not found in the non-ASD group. These findings may also indicate that ASD children need to recruit domain-general cognitive abilities differently from their non-ASD peers in order to engage in mathematical problem solving. Based on this, it could be hypothesized that the use of simplistic strategies to solve mathematical problems by ASD children is indicative of a lower cognitive profile in these functions, which could help to identify the subgroup of ASD children with the most mathematical difficulties.

Regardless of the level of abstraction used, results also support differences in the cognitive profiles between children in both groups. Thus, ASD children who exhibited poorer mathematical performance (i.e., ≤ 25% of correct responses) showed comparatively lower scores in inhibition, ToM and verbal comprehension, whereas such association was not found in the non-ASD group with poor performance. Overall, these findings support lower scores in certain components of executive functions and lower ToM scores within the ASD group with low performance in mathematical problem solving. They also indicate that the level of verbal comprehension in ASD children could be another variable involved in the resolution of mathematical problems, in line with previous works (Alderson-Day, 2014).

Some studies have already shown that the strategies and representations used by ASD children are variables that determine their performance (Bruno et al., 2021; Polo Blanco et al., 2021; Polo-Blanco & González-López, 2021; Polo-Blanco et al., 2022). Others have shown that some of the cognitive traits intrinsic to the disorder, such as low executive functioning profile, can directly interfere with mathematical performance and with implementing the actions that are needed to solve mathematical problems (Bull & Scerif, 2001; Kim & Cameron, 2016). In particular, working memory has been found to be a predictor of individual differences in problem solving and computation development in ASD children (Bullen et al., 2020; Chen et al., 2019). Also consistent with our findings, sentence comprehension and mathematical vocabulary have been associated with skills for solving mathematical problems in non-ASD children (Bae et al., 2015). Finally, our results show a relationship between lower ToM scores and difficulties in problem solving (in terms of both, strategy used and accuracy) in ASD children, a novel aspect that, to the best of our knowledge, had not been studied in this population.

Previous studies have also delved into these variables in the typically developing population (Kintsch & Greeno, 1985; Lee et al., 2009; Viterbori et al., 2017), and likewise identified executive functions as predictive variables of performance for solving mathematical problems. Within the executive functions, working memory is the variable that most determines performance when solving mathematical problems (Bull & Scerif, 2001; Bull et al., 2008; Gathercole et al., 2004; Keeler & Swanson, 2001; Swanson & Beebe-Frankenberger, 2004) and it has more of an influence on mathematical competence than the combination of cognitive inhibition and flexibility factors (Lee et al., 2013; Monette et al., 2011). ToM has also been associated with mathematical competence in the neurotypical population (Lecce et al., 2014), and it has been claimed to be particularly important in mathematical tasks that involve reasoning and choosing an effective strategy, especially when the tasks are presented verbally (Lockl et al., 2017).

In contradiction with these findings, in our study we did not find any association between performance in problem solving and these cognitive variables in the non-ASD group, independently of their performance, which would suggest that the difficulties solving mathematical problems of the non-ASD children could have a different nature than in the ASD population, and thus involve different cognitive variables. However, the absence of positive findings in the non-ASD group could also be explained by a small sample size or by methodological differences when measuring the cognitive variables or mathematical performance compared to other studies (Cantin et al., 2016; Lockl et al., 2017). Future studies would be necessary to clarify and examine these aspects.

Our results provide valuable information to help our understanding of mathematical problem-solving difficulties in ASD children. In particular, they have direct implications on the design of educational interventions in ASD children with mathematical difficulties. For instance, as seen in other mathematical contexts, ASD children may not acquire advanced strategies spontaneously, as non-ASD children often do, so they may benefit from explicit instruction in strategy use (Polo-Blanco & González-López, 2021). Interventions should also consider stimulating the cognitive functions involved in mathematical problem solving that are most affected in ASD population (cognitive flexibility, inhibition, ToM and verbal comprehension) (Westby & Robinson, 2014; Whalon & Cox, 2020). Some evidence-based strategies for children with learning difficulties, such as Schema Based Instruction (SBI) (Fuchs et al., 2004) or the Conceptual Model-Based Problem Solving (COMPS) approach (Xin, 2018), have been successfully adapted to ASD children’ characteristics improving their ability to solve mathematical problems (Bruno et al., 2021; García Moya et al., 2022; Polo-Blanco & González-López, 2021; Polo-Blanco et al., 2021, 2022; Root et al., 2017). Future empirical studies are needed to measure the effects of these adaptations on the development of some of the cognitive functions with lower scores found in ASD children.

It should be noted that this study is subject to some limitations that must be considered when interpreting the results. First, the relatively small sample size limits the statistical power and external validity of the results. Second, although one of the inclusion criteria for the ASD and comparison groups was not presenting intellectual disability (i.e., having an FSIQ ≥ 70), the ASD sample presented a significantly lower FSIQ than the non-ASD one. This leads us to think that it may be necessary to match children in both groups by FSIQ intervals, as well as by age, sex, grade level and school, as it was done. To try to minimize this, the FSIQ (as continuous variable) was considered as a covariate in all our analyses; additionally, stratified analyses by FSIQ were explored, and main multivariate analyses were performed in the group with average FSIQ (90–109). However, the applicability of the stratification by FSIQ should be taken with caution, given the small sample size of each level. Third, despite using a large neurocognitive battery, working memory was not dichotomized into the visual-spatial and auditory subtypes, which could have yielded additional results. However, as strengths, we must highlight the requirement of absence of comorbidities in the ASD group, and the inclusion of a non-ASD group matched by sex, age and school (grade and classroom). Moreover, to our knowledge, this is the first study to analyze the relationship between the types of strategies used to solve mathematical problems, in terms of level of abstraction, the accuracy rate of responses and neurocognitive variables in ASD and non-ASD children. Finally, it should be noted that since many of the studies cited in this paper have been conducted with English-speaking children, some of the results should be interpreted with caution. Given that the language of instruction or task language might impact mathematical thinking and learning (Schleppegrell, 2007), more studies in different languages are needed to assess the impact of each language individually on students' mathematical performance.

In conclusion, there appears to be a relationship between some cognitive functions in ASD children and mathematical performance in problem solving. Specifically, lower scores in inhibition, verbal comprehension and ToM seem to be associated with poorer mathematical performance; furthermore, there appears to be a positive correlation between cognitive flexibility, inhibition and ToM and the strategies used to solve mathematical problems.

It is essential to deepen the understanding of the nature of the apparent low mathematical performance in the ASD population and its causes. For example, multicenter controlled studies could be carried out with a larger sample size to differentiate subgroups of ASD children and identify different neuropsychological profiles. This would allow more conclusive results that would help in the design of intervention strategies to improve performance and accessibility to greater educational opportunities in this population.
